# Practices of Informed Consent for Emergency Procedures at a Tertiary Care Hospital in Lahore, Pakistan

**DOI:** 10.7759/cureus.50322

**Published:** 2023-12-11

**Authors:** Tayyba Munawar, Fiza Ismail, Haseeb Mehmood Qadri, Amjid ul Haq, Ali Munawar, Abdul Subhan Zahid, Muhammad Sheraz, Muhammad Saad Babar

**Affiliations:** 1 General Surgery, Lahore General Hospital, Lahore, PAK; 2 General Surgery, Shaikh Zayed Hospital, Lahore, PAK

**Keywords:** emergency, trauma, audit, pakistan, practice, surgical informed consent, patient consent

## Abstract

Background

The purpose of obtaining informed consent is to ensure that patients undergoing any medical or surgical intervention are neither deceived nor coerced. Accurately estimating surgical risks is critical for shared decision-making and informed consent. Probable complications and alternative procedures should be presented to the patient so that they can freely choose an operative option. However, this factor is difficult to carry on in emergencies where an urgent decision is required.

Objective

This study aimed to assess the ongoing clinical practices of informed consent in emergency surgeries at a tertiary care facility.

Materials and methods

A cross-sectional survey was carried out from March 2022 to June 2022 at the Department of General Surgery, Lahore General Hospital, Lahore, Pakistan, with patients who had undergone surgical procedures under local, spinal, or general anesthesia within 24 hours of presentation. A Google Form (Google Inc., Mountainview, CA) was designed, containing a predefined set of 32 standard questions, and patients were interviewed in their native language to assess their satisfaction regarding the pattern and components of emergency informed consent. Categorical data were assessed using measures of central tendency, frequencies, and percentages.

Results

A total of 169 patients were selected for the study. Only 1.6% of them signed the consent form themselves, while 93.5% of the forms were signed by their first-degree relatives. Verbal consent was taken in 4.8% of cases. In 88% of cases, informed consent was obtained by the house surgeons. The majority of patients, i.e., 78.2%, were not able to read the written consent form; however, 83.1% understood the verbal information. About 66.3% of patients agreed that they were informed about the nature of their disease, while 67.5%, 14.8%, and 13.7% affirmed that they were explained the nature of surgical intervention, associated risks, and type of anesthesia, respectively. Overall, 59.5% of patients felt satisfied with the process of informed consent. About 91.1% of the patients believed that their decisions were unaffected by the procurement of informed consent.

Conclusion

The existing practices of informed consent and comprehension by the population were found to be substandard. Physicians seem to ignore bioethics, and patients appear to be unaware of their basic rights. Although practiced at our center, not all components of informed consent were communicated to the patients. The risks of the procedures and the mode of anesthesia used were not well addressed by doctors. There is a grave need to educate the medical community about the legal and ethical aspects of informed consent, as well as the public masses regarding their rights.

## Introduction

Informed consent can be defined as a tool to educate and inform a competent patient about their disease, the risks and benefits of the intervention being chosen, and alternative options so that they can make an informed decision about their health [1,2]. A properly designed informed consent includes a) autonomous authorization; b) disclosure; c) the capability of the patient to understand; d) comprehension; and e) consent while fulfilling both ethical and legal aspects of medical excellence [3, 4]. Appropriate consent solidifies the patient-clinician relationship of trust, provides the patient with a sense of autonomy and shared decision-making, and ensures the protection of the physician and his institute against possible legal implications [3]. Ways to obtain consent may vary due to cultural ramifications in low- and middle-income countries (LMICs) that may include surrogate consent, community consent, and waived consent [5]. A surrogate is a patient’s legally accepted representative, while community consent allows community leaders to make decisions [5].

Over the years, with improvements in many other professions in Pakistan, informed consent has become an area of medical practice that has yet to be improved [6]. Consent, in its true form, is both an oral discussion and a written document. It should give mutual satisfaction to both parties involved. However, in Pakistan, limitations occur due to different factors like low socioeconomic status, patriarchal family structure, and a lack of awareness leading to exploitation [3]. Risks are generally vaguely described, maintaining the positive perspective and incorrect documentation on consent forms that lead to invalidation of consent and put the medical team at risk of medico-legal consequences [7, 8]. This study was done to assess informed consent practices in emergency surgical procedures at a public-sector tertiary care teaching hospital [8].

## Materials and methods

This cross-sectional, prospective audit was conducted from March 1, 2022, to June 30, 2022, for four months at the Department of General Surgery, Lahore General Hospital, Lahore, Pakistan, after obtaining approval from the Departmental Review Board (approval number: SU-III/74/LGH, dated April 1, 2022).

Inclusion criteria

Patients of any age, irrespective of their gender, who had undergone any surgical procedure under local, spinal, or general anesthesia and patients who presented to the emergency room on their first presentation were included in the study.

Exclusion criteria

All unattended patients and patients who were referred from the outpatient department were excluded.

Setting of data collection

Permission was obtained from the participants before the interviews after explaining the purpose of the interview. There was consecutive recruitment of patients presenting for emergency surgery. Data were collected from patients and guardians of children using questionnaire-based, semi-structured interviews conducted 24 hours after general anesthesia, 12 hours after spinal anesthesia, and two hours after local anesthesia when the patients had fully recovered from the effects of anesthesia, were comfortable and willing to participate, and had a Glasgow Coma Scale (GCS) score of 15/15.

Instruments of data collection

A bilingual Google Form (Google Inc., Mountainview, CA) containing a predefined set of 32 standard questions in English and Urdu was developed from the existing scientific literature, and patients were asked to respond on demographic details, pattern of emergency informed consent, components of emergency informed consent, and feedback for the whole process of consent.

The interviews were conducted by the authors, who were first trained for the purpose, and they explained the questions to the subjects. The data were analyzed using a Microsoft Excel (Microsoft Corp., Redmond, WA) spreadsheet. Categorical data were assessed using measures of central tendency, frequencies, and percentages.

## Results

**Figure 1:**
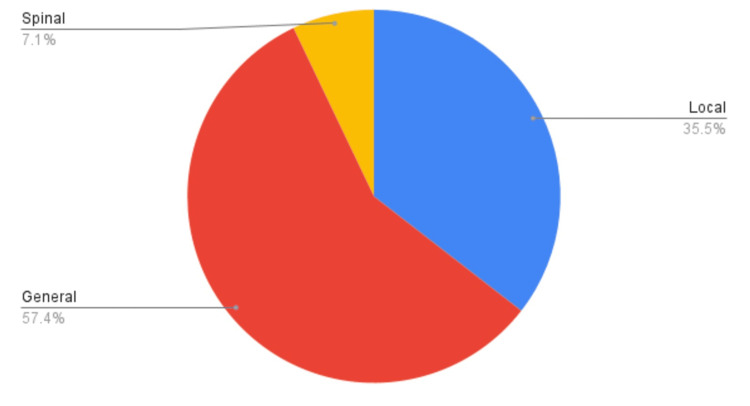
The type of anesthesia given in various procedures for the included patient population in emergency operation theaters.

A total of 169 patients were included in the study, out of which 68.6% were male and 31.40% were female. Approximately 57.4% of cases were done under general anesthesia, 35.5% under spinal anesthesia, and 7.1% under local anesthesia (Figure 1).

**Figure 2:**
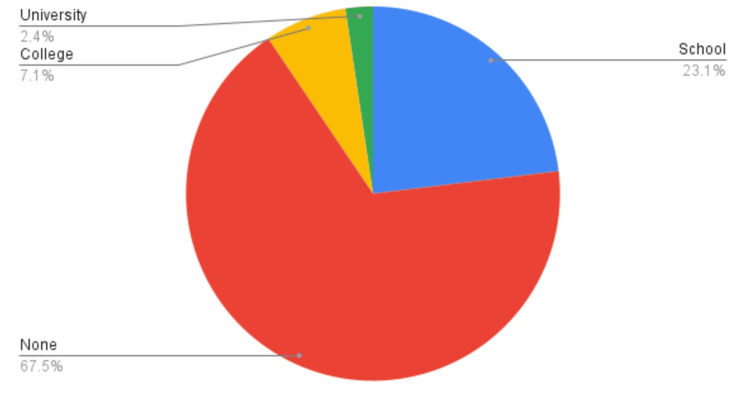
The educational status of the included patient population.

Literacy was assessed for each patient being interviewed. Around 23.1% of affected patients had been educated in a school, while 67.5% of patients had never attended an educational institution in their life (Figure 2).

From 71.4% of participants, consent was taken before the procedure, while 28.6% of operated patients confessed that consent was not taken from them or their attendants. In 91.1% of cases, written consent was taken, and in 8.9% of cases, verbal consent was taken. House surgeons obtained consent in 88% of cases, while residents, surgeons, and nurses obtained consent in 6.4%, 4.6%, and 1% of cases, respectively. In 93.5% of cases, relatives of patients signed the document; in 4.9% of cases, written consent was not obtained; and in 1.6% of cases, patients signed the document themselves. In 96.7% of cases, consent was obtained just before the surgery; 83.1% understood the verbal information; 78.2% carefully read the document; and only 23.4% understood all the written information (Table 1).

**Table 1:** Pattern of obtaining consent from the patients

S. No.	Pattern of obtaining consent	Options	Results (%)
1.	Was your consent taken before the procedure?	Yes	71.4%
		No	28.6%
2.	How was the consent obtained?	Verbal	8.9%
		Written	91.1%
3.	Who obtained the consent?	Surgeon	4.6%
		Resident	6.4%
		House officer	88%
		Nurse	1%
4.	Who signed the consent document?	Patient	1.6%
		Relative	93.5%
		Verbal only	4.9%
5.	When was the consent obtained?	Just before surgery	96.7%
		During surgery	3.3%
		After surgery	None
6.	Did you understand the verbal information?	Yes	83.1%
		No	16.9%
7.	Did you read the document carefully?	Yes	21.8%
		No	78.2%
8.	Did you understand all the written information?	Yes	23.4%
		No	76.6%

The nature of the disease was explained in 66.3% of cases. The proposed treatment was discussed in 67.5% of cases. Alternate treatment options were discussed in 90.5% of cases. The type of anesthesia was discussed in 86.3% of cases. In 90.5% of cases, participants were encouraged to ask questions. Informed consent influenced the decision of 89.9% to opt for surgery. Among the study population, 87.6% of participants were in favor of the process of informed consent, and only 59.5% were satisfied with the whole process of consent-taking (Table 2).

**Table 2:** Components of consent-taking and feedback from patients on informed consent.

S. No.	Questions regarding the components of informed consent	Results (%)	
		No	Yes
1.	Was the nature of the disease explained?	33.7%	66.3%
2.	Was the proposed treatment discussed?	32.5%	67.5%
3.	Were the alternative treatment options discussed?	90.5%	9.5%
4.	Were the risks of the proposed treatment explained to you?	85.2%	14.8%
5.	Were the benefits of the proposed treatment explained to you?	79.9%	20.1%
6.	Was the type of anesthesia discussed?	86.3%	13.7%
7.	Were the risks of anesthesia discussed?	95.8%	4.2%
8.	Were the benefits of anesthesia discussed?	97%	3%
9.	Were you encouraged to ask questions?	90.5%	9.5%
10.	Did the informed consent influence your decision to go for surgery?	89.9%	10.1%
S. No.	Feedback from the patients on informed consent	No	Yes
11.	Are you in favor of the process of informed consent?	87.6%	12.4%
12.	Were you influenced by anyone to proceed with surgery?	91.1%	8.9%
13.	Were you satisfied with the whole process of consent-taking?	59.5%	40.5%

## Discussion

Informed consent refers to the voluntary agreement of a competent individual to the nature, potential risks, benefits, and alternative options related to an intervention [9]. Consent is an exigent ethical issue in emergency care in LMICs [5]. It is a well-established concept that when human subjects are invited to participate in both clinical and research settings, it necessitates the requirement of informed consent [3]. In the West, we have seen a shift from a paternalistic model of practice to a more patient autonomy-centered approach, yet the idea of informed consent being involved in patient autonomy is unknown to both patients and physicians in our society [3, 7].

In this study, it was observed that 71.4% of the patients expressed informed consent, 91.1% of which documented it in written form. However, a substantial divergence was noted: a striking 96.7% of the consents were produced immediately before the surgeries, in contrast to the mere 22% mentioned in the literature [4]. This overwhelming amount of information might be difficult for a patient to comprehend in this limited time [6]. As the patient becomes emotionally and psychologically dependent on the healthcare provider, the urgency to make a quick decision can threaten the validity of informed consent [5].

In this study, 90% of the patients got no information about other available treatments. Moreover, 85.2% of the patients were not informed of potential risks, while 79.9% were not briefed on the benefits of the intervention to be done. The nature of the disease and the proposed treatment options were discussed with more than 60% of the patients, comparable to the findings in a study by Siddiqui et al., which stated that 87% of the patients received information regarding their disease and 70% were informed about the nature of the surgical procedure [1]. A study on surgical malpractice claims found that most of the claims filed were related to postoperative complications, where patients felt that they were not properly informed about potential complications and outcomes [10].

One of the reasons that leads to less information for the patients is that mostly the physicians and researchers themselves are part of a family-based, hierarchical, and patriarchal society, which sometimes leads to ignorance of bioethics [7]. According to a survey, most general practitioners did not consider it necessary to explain the details of the treatment advised, even knowing that the patient has a right to know. This was further corroborated by the patient's reluctance to receive bad news [7]. This is in contrast to a Pakistani audit, which suggests that none of the patients considered the explanation of risks as a reason to reconsider surgery [1].

The majority of the documents (93.5%) were signed by a relative of the patient, while only 1.6% were signed by the patient, in contrast to another study conducted at private hospitals in Pakistan in which 42% of the consents were signed by the patients [4]. In LMICs, surrogate consent is taken predominantly as compared to waived consent [5]. In Pakistan, families seem to have a more influential role in decision-making along with patients during treatment, and the participation decision is usually influenced by factors such as poverty, illiteracy, and an oppressive mindset [3, 7].

In the current study, 67.5% of subjects had no educational background. High levels of illiteracy and the inability of patients to understand the local and official languages led to unsatisfactory results [7]. This study shows that 90.5% of the cases were not encouraged to ask questions, similar to another local study in which 77% were not encouraged to ask questions as well [4]. This is in contrast to another national study conducted at a private hospital, where 93% of the patients were allowed to get their questions answered. Despite the patients being educated, they were unable to understand medical terminology [11]. We propose that this difference in percentages may be attributed to the fact that private-sector hospitals have a lower patient turnout than public-sector hospitals, implying less burden and more efficiency for doctors.

This study shows that 88% of consents were taken by house surgeons, while only 4.8% of consents were taken by primary surgeons. The majority of patients in this study understood the verbal information, while more than 70% of patients were unable to read and understand the written information on the consent form, which is similar to another study where 54.7% were not able to understand the information provided, citing the reasons as discussed in non-native language (74.5% responses) and consent being taken by junior doctors who were not well-versed with the diagnosis or planned procedure (12.2%) [1]. Another study explained the same results, which are in contrast to best practices in the developed world [4]. The process of obtaining consent is often carried out by junior doctors, which may lead to incorrect documentation, placing both consultants and trainees at risk of medico-legal consequences. It also suggests a need for discussion with consultant surgeons as to what information should be included on consent forms [8, 12].

Only 13.7% of the patients were briefed on the types of anesthesia, not explaining the benefits and risks to more than 90% of patients. This is in striking contrast to a study in which types of anesthesia were explained to 100% of patients [4]. Another study explained that 66% of the patients received information about anesthesia, and none were explained the risks and consequences associated with anesthesia since the surgeons are usually unaware of the details of the anesthesia given. Therefore, it is usually considered the duty of anesthetists to explain all the information [1]. Additionally, a field-specific discussion of risks versus benefits is essential on the part of anesthetists as well as operating surgeons [13].

The satisfaction rate in this study was 59.5%. A higher satisfaction trend is seen among patients if the surgeon shares important and relevant information before surgery, making them feel involved even if they are unable to comprehend the given information. Due to a lack of governance and an effective local government, local ethics and the ethical conduct of research fall on the investigator. The literature does not explain any guidelines for ethical emergency care research in LMICs. Thus, to make a study ethical, it must demonstrate an acceptable risk-benefit profile, yet the literature does not explain what risks are acceptable in emergency care [10].

Limitations of the study

The emergency context of the audit is a critical limitation. In emergencies, obtaining informed consent becomes challenging due to the urgency of the situation. This will definitely affect standards. The general level of awareness regarding informed consent among the public could have provided context to some of the findings. The reason for consent being signed by first-degree relatives could have been studied in depth, i.e., the patient being too ill to sign, social or cultural bias, etc.

Clinical recommendations and future implications

Awareness should be raised among patients to make them aware of their condition, the treatment process, and the prognosis. On the other hand, doctors should also be compelled to provide patients with their rights. Ethically, it is also the responsibility of every healthcare worker to make things easier for the sufferers. A doctor is required to obtain the patient’s consent before continuing if the patient is conscious, alert to time, place, and people, and intellectually capable of comprehending the information regarding his ailment and treatment.

Literature suggests that the process of informed consent can be improved and standardized by teaching ethical and legal aspects using modern tools such as e-health and multimedia programs. A better method of obtaining consent can be accomplished on a study-by-study basis by establishing literature that assesses the quality of consent, understanding barriers to consent, community governance, structure, and whether the consent model is being appropriately used, which will help tailor an approach appropriate to culture [10, 14]. A solution to this problem could be the standardization of pre-printed emergency consent forms, which may significantly improve documentation, enhance informed consent, and increase compliance with guidelines [8, 15]. This is especially important in an emergency setting because patients in an emergency are more inclined to experience perioperative complications than patients going for elective surgery [2]. Systematic reforms shall be implemented, such as periodic training of professionals focused on patient communication and employing the use of technology to aid with the consent process, for example, using explanatory videos for complex concepts [16].

Informed consent can be influenced by many psychological and sociocultural variables that can influence patient understanding. Further studies and audits can focus on these factors and understanding the barriers that can aid in strategizing ways around them.

## Conclusions

The findings of the subsequent audit revealed that postgraduate residents had neglected to include the key elements of informed consent. Most often, the house surgeons, who were not trained to take valid informed consent by explaining all the required information to the patients, took the patients’ consent. Another major problem was that the consent was taken from patients’ first-degree relatives, which caused the most damage. Lack of literacy and lack of interest in rights for one's self on the patients' part and lack of bioethics on the part of doctors are clear areas of malpractice in informed consent.

There were interventions done at our center post the audit. A lecture on bioethics was conducted for postgraduate residents, describing the results of the audit, patterns, and components of informed consent. We aim to conduct a re-audit to evaluate the clinical practices of informed consent in emergency procedures in the future.
